# Measurement and Analysis of Microwave Frequency Signals Transmitted through the Breast

**DOI:** 10.1155/2012/562563

**Published:** 2012-03-07

**Authors:** Jeremie Bourqui, John Garrett, Elise Fear

**Affiliations:** Department of Electrical and Computer Engineering, Schulich School of Engineering, University of Calgary, Calgary, AB, Canada T2N 1N4

## Abstract

Microwave approaches to breast imaging include the measurement of signals transmitted through and reflected from the breast. Prototype systems typically feature sensors separated from the breast, resulting in measurements that include the effects of the environment and system. To gain insight into transmission of microwave signals through the breast, a system that places sensors in direct contact with the breast is proposed. The system also includes a lossy immersion medium that enables measurement of the signal passing through the breast while significantly attenuating signals traveling along other paths. Collecting measurements at different separations between sensors also provides the opportunity to estimate the average electrical properties of the breast tissues. After validation through simulations and measurements, a study of 10 volunteers was performed. Results indicate symmetry between the right and left breast and demonstrate differences in attenuation, maximum frequency for reliable measurement, and average properties that likely relate to variations in breast composition.

## 1. Introduction

Microwave approaches have been proposed as complementary methods for breast imaging, providing images related to the electromagnetic properties of tissues in the breast. Significant variations in the properties of healthy tissues are expected, leading to challenges in both measurement of microwave signals and creation of meaningful images. A recent study analyzed measurements of 354 samples of healthy breast tissues that had been surgically excised [1]. Results indicated that permittivity and conductivity increased with decreasing proportion of adipose tissues in the samples. Specifically, fatty tissues are expected to exhibit low permittivity and conductivity at microwave frequencies, while the properties of glandular tissues are expected to be several times greater. A second study analyzed properties of malignant tissues, suggesting significant differences in the properties of these tissues when compared to fatty tissues and a much less dramatic difference (on the order of 10%) when comparing malignant and glandular tissue properties [2]. The composition of the breast also varies from patient to patient. For example, women may have primarily fatty tissues in the breast or dense breasts that consist primarily of glandular tissues. The combination of breast composition and electromagnetic properties of breast tissues suggests that microwave imaging involves detecting small changes in a complex environment. Further more, challenges related to microwave imaging and measurement are also anticipated to vary with breast composition, as lower attenuation of signals and high contrast between healthy and malignant tissues are expected for fatty breasts.

In order to gain insight into the challenges related to measurement and imaging, simulation models based on breast MR scans of patients have been developed [3]. These models provide realistic breast shapes and spatial distributions of tissues. In attempt to accurately represent tissues properties, dielectric properties models that are based on the measurement study are incorporated [4]. To enhance the match between simulated and measured data, models of the antennas and aspects of the system (e.g., immersion liquid) are also included. Simulations of the breast models and sensors performed with numerical techniques such as the finite difference time domain (FDTD) method provide insight into reflected and transmitted signals expected in measurement. For example, a recent study compared measurements of volunteers and simulations of patient-specific models, showing good agreement between the dominant reflections [5]. However, differences between simulations and measurements of later-time reflections were evident, and transmitted signals were not measured. Transmitted signals are of particular interest in light of another recent study of the properties of breast tissues, which suggests differences between *in vivo* and *ex vivo *measurements [6]. If differences between reported measurements and actual tissue properties exist, then simulation models may not accurately predict signals transmitted through the breast. This information is a valuable tool for specifying requirements for measurement systems, such as dynamic range and frequency band.

Several prototype systems have been designed for microwave breast imaging and include collection of transmitted signals. For example, one system for microwave tomography has an array of antennas positioned at distance from the breast and placed in a lossy immersion liquid [7]. This system is designed to operate from 500 MHz to 3 GHz. A second example, a multistatic radar system, has antennas arranged on a hemisphere [8]. A dielectric insert and layer of immersion liquid are used to separate the antennas from the breast. The bandwidth for this system is in the range from 4 to 10 GHz [9]. Although both systems collect transmitted data, the signals are expected to be influenced by the external environment rather than predominantly effected by the breast. Different immersion liquids are also expected to have different effects on the transmitted signals that are related the electromagnetic properties of the liquids. In addition, both systems have been designed for a specific frequency band of operation and do not permit the assessment of transmission over a very wide frequency band including lower frequencies.

Therefore, there is a need to assess the upper frequency limit at which signals transmitted through the breast may be reliably measured and also to gain insight into intra- and inter-patient differences in this maximum frequency. In this paper, we report a system designed to measure signals transmitted through the breast at frequencies in the range of 1–10 GHz. The sensors are in direct contact with the breast and a lossy immersion liquid is employed to attenuate alternative paths that the signals may travel. Measurement of transmitted signals with known separations between sensors and without significant path in the coupling medium also provides the opportunity to assess the average properties of breast tissues. Reflection-based measurements contain too much ambiguity to make an assessment of average properties practical (using a limited number of sensor locations). We further note that this system is not designed explicitly for imaging, although estimation of average properties of breast tissues would be helpful for microwave imaging algorithms. After validation of the system, a study of 10 volunteers is performed in order to gain insight into the changes in attenuation and maximum frequency of operation that occur for different patients and within the same patient. We report a simple technique to estimate the average permittivity and conductivity of the tissues from transmission measurements and apply this technique to the measurements collected from patients.

## 2. Methods

In this section, the system to measure microwave signal transmission through the human breast and approach to average property estimation are described. The electrical conductivity of breast tissues is expected to vary significantly between patients, which translates to variations in signal attenuation. Cases, in which very high attenuation occurs, (e.g., predominantly glandular tissues) are expected to lead to measurement challenges due to extremely weak transmitted signals. These challenges have to be addressed by careful design of two aspects of the measurement system. First, the overall dynamic range needs to be as high as possible. Secondly, there are multiple paths that the signals may take; the signals that travel through the breast are of interest, while the signals that travel around the breast are not. Therefore, the system must facilitate detection of signals traveling through the breast only. The sensor design and system configuration implemented to achieve these goals are described in Section 2.1. The system also provides an opportunity to gain additional information through estimating average properties of the breast tissues. A simple approach to roughly estimating the average properties is discussed in Section 2.2.

### 2.1. Sensor and System

**Figure 1:**
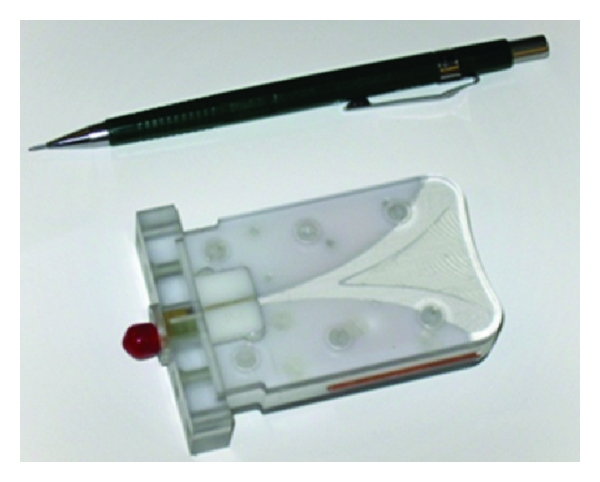
Cassiopeia antenna.

**Figure 2:**
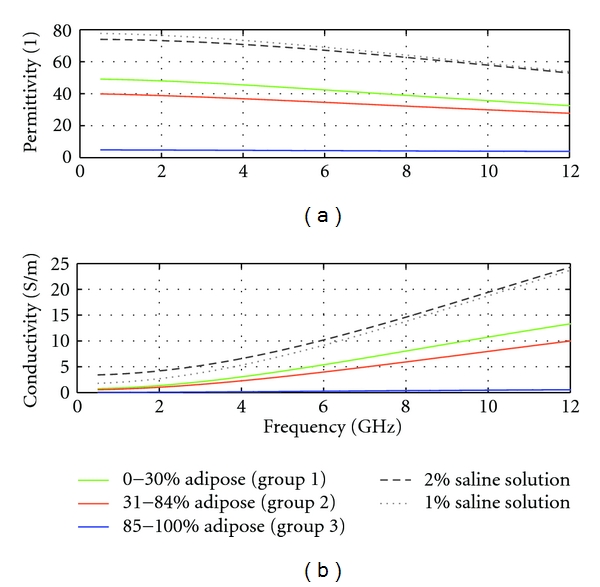
Relative permittivity and conductivity of the 3 breast tissue groups, as well as 1% and 2% saline solutions.

The Cassiopeia antenna (Figure 1) [10] is the sensor used to both transmit and receive signals. This antenna is designed to come into contact with the breast skin and to operate in a lossy immersion liquid. The antenna is essentially a balanced antipodal Vivaldi antenna (BAVA) with a director included in the aperture, similar to the antenna reported in [11]. The key differences are a modified feed structure and the use of higher permittivity materials to confine fields to the antenna such that the sensor is relatively insensitive to the immersion liquid. The details of the antenna and feed design, along with initial simulation and measurement results with the Cassiopeia placed in air and glycerin, are found in [10]. For this study, the antenna is placed in a 2% saline solution in order to attenuate signals that travel along paths outside of the breast. As shown in Figure 2, the 2% saline exhibits conductivity well above the expected conductivity of breast tissues, which significantly attenuates signals traveling along these unwanted paths.

**Figure 3:**
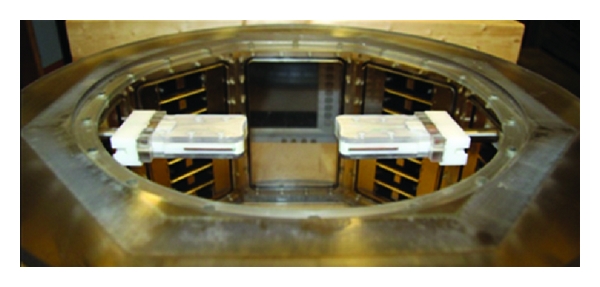
View of the measurement tank with the two sensors (Cassiopeia antenna) attached to movable arms.

**Figure 4:**
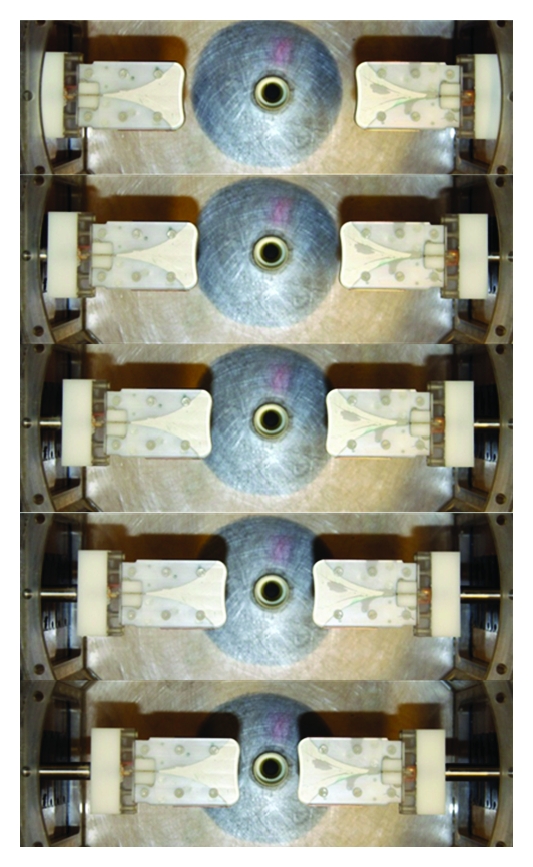
View of the sensors set at different separation distances.

To collect transmission measurements, two sensors are placed on opposite sides of the tank (or breast) (Figure 3). The sensors are placed 37 mm below the top of the tank in order to completely immerse the sensors in the saline. The two sensors are aligned, and each sensor is mounted on a sliding arm, as shown in Figures 3 and 4. The separation distance can be manually adjusted between 140 mm and 10 mm using a knurled wheel; digital callipers are attached to both sides so that precise separation distances can be recorded. This configuration permits the antennas to be positioned such that contact is made with the breast skin. Modifying the separation distance may easily be accomplished with high accuracy; several measurements at different separation distances are typically collected. The antennas are connected to a vector network analyzer (PNA N5242A, Agilent Technologies, Palo Alto, CA, USA). To maximize sensitivity, measurements are performed with an intermediate frequency (IF) bandwidth of 10 Hz and a port power level of 10 dBm. These settings produce a comfortable 120 dB dynamic range at the antenna ports without need for additional averaging. A total of 401 points are recorded over a 1-to-10 GHz bandwidth. The total sweep time is 36 seconds.

**Figure 5:**
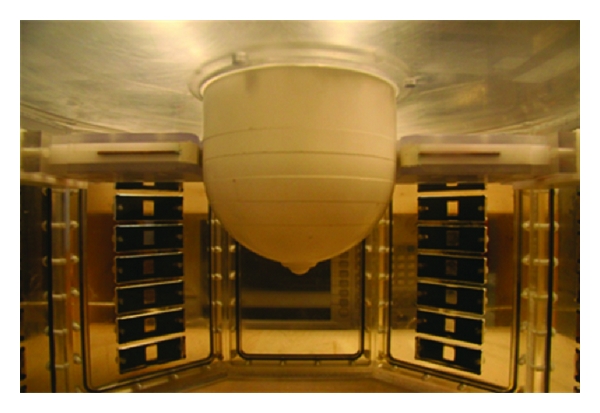
View of the low-loss breast model in the measurement tank. Picture was taken without the 2% saline for better clarity.

The performance of the sensor in saline and the ability of the sensors to measure transmitted signals are assessed. First, measured and simulated reflection coefficients over a broad-frequency band are inspected. Simulations are also performed to evaluate the integrity of the radiated field distribution in breast tissues. The breast model examined consists of a 2 mm thick skin layer filled with homogeneous breast tissue representing fat (tissue group 3 which represents median properties of samples containing 0–30% fat in [4]). The dimensions of the breast model are detailed in [11]. An FDTD simulation tool (SEMCAD X, SPEAG, Zurich) is used to perform the simulations with parameters similar to those reported in [10]. Dispersive model parameters published in [4, 12, 13] are used to model the 2% saline, skin, and tissue group 3, respectively. Validation of the radiated fields through measurements is challenging, so transmitted signals are measured through breast models representing low and high loss scenarios. The hemispherical, low-loss breast model is described in [14] and shown in Figure 5. This model has a diameter of 100 mm and is made of a solid dielectric material with relative permittivity of 15 and loss tangent supposedly less than 0.002 (Eccostock HiK, Emerson, and Cuming Microwave Products, Randolph, MA, USA). The high-loss breast model has the same dimensions, however, consists of a thin rubber membrane filled with a 1% saline solution. The conductivity of 1% saline is greater than the conductivities reported for the three breast tissue groups in [4] but below the 2% saline, thus very weak transmission is expected.

**Figure 6:**
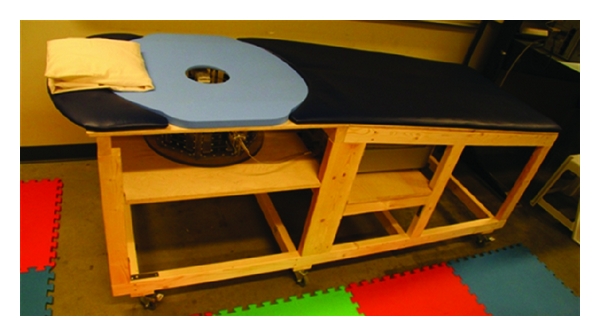
Measurement system integrated in the user interface.

Figure 6 shows the user interface used for work with human participants. It consists of a bed on which the volunteers lie in a prone position. The bed includes an opening where the breast extends into the measurement tank placed underneath. The PNA is located under the bed directly behind the measurement tank. Several measurements are collected from each volunteer (more details on the volunteer study are provided in Section 4). The antennas are placed in contact with the breast, and an initial measurement is collected. The antenna separation is modified, and a new set of measurements is obtained. This process is repeated several times, resulting in a set of transmission coefficients collected with different separation distances between the antennas. Note that the full scattering coefficient matrix (*S*_11_, *S*_21_, *S*_12_, and *S*_22_) is recorded for each measurement.

### 2.2. Property Estimation

A simple method to estimate the average electrical properties of the breast tissues is introduced. The method takes advantage of the differences between transmission coefficients recorded at two different separations between the antennas, however, involves several assumptions. First, it is assumed that the contact between the skin and the sensor remains unchanged after repositioning one of the sensors. Secondly, the change in response due to cable movement is considered negligible. These assumptions are believed to be reasonable based on the stability of the reflection coefficients (*S*_11_ and *S*_22_) observed between measurements. Third, it is assumed that the inner structure of the breast is the same for two measurements collected at different separations (i.e., the two paths have the same average properties). Therefore, the difference between the transmitted signals is assumed to be solely related to the change in transmission length induced by reducing the separation between the sensors. To relate the change in transmission coefficient to the average electrical properties of the breast tissues, we further assume a uniform plane wave model to describe the propagation from the transmitting to receiving antenna. This implicitly ignores any effects of multipath in the breast tissues. The electric field in the tissues is approximated as:

(1) $$E^{+}\left(z\right)=E_{0}\cdot e^{-\alpha \cdot z}\cdot e^{-j\cdot \beta \cdot z},$$

where *α* is the attenuation constant, and *β* is the phase constant. These constants are then approximated using a pair of transmission measurements along with a correction for radial spreading of the signal:

(2) $$\begin{array}{c} \alpha \left(f\right)=\frac{-\log\left({\left|S_{21}\left(f\right)\right|}_{1}/{\left|S_{21}\left(f\right)\right|}_{2}\right)\left(D_{1}/D_{2}\right)}{D_{1}-D_{2}},\\ \beta \left(f\right)=-\frac{\theta_{1}\left(f\right)-\theta_{2}\left(f\right)}{D_{1}-D_{2}}, \end{array}$$

where *D*_*i*_ is the separation distance at which the measurements has been taken, |*S*_21_(*f*)|_*i*_ is the magnitude of the transmission coefficient and, *θ*(*f*)_*i*_ is the phase of the transmission coefficient. Subscripts 1 and 2 refer to the two measurements used for the calculations. Note that *D*_1_ must be greater than *D*_2_.

After the attenuation and phase constant are retrieved, the corresponding permittivity and conductivity are calculated using (3):

(3) $$\begin{array}{c} \varepsilon_{r}\left(f\right)=\frac{{\beta (f)}^{2}-{\alpha (f)}^{2}}{{\left(2\pi f\right)}^{2}\mu_{0\;}\varepsilon_{0}},\\ \sigma \left(f\right)=\frac{2\alpha \left(f\right)\beta \left(f\right)}{2\pi f\mu_{0}}, \end{array}$$

where *ε*_0_ and *μ*_0_ are the permittivity and permeability of vacuum.

## 3. Validation

The measurement system is validated by first examining the behaviour of a single sensor, then through collecting transmission data. More specifically, the ability to adequately measure transmitted signals over the entire frequency band of interest and the feasibility of the property estimation method are explored.

### 3.1. Sensor and System Performance

**Figure 7:**
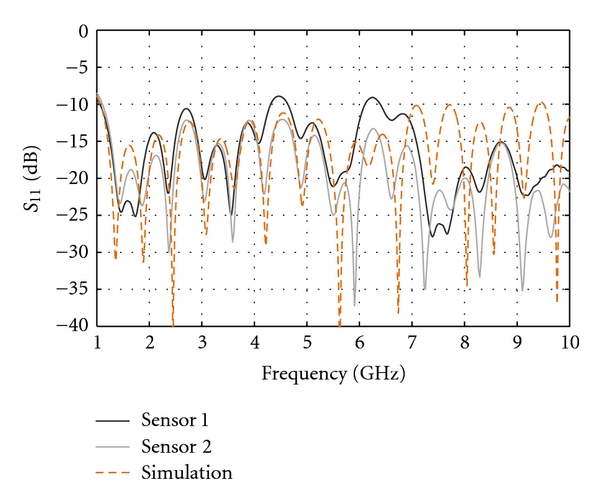
Reflection coefficient measured for both sensors when immersed in 2% saline. Simulated counterpart is in dashed line.

Figure 7 presents the reflection coefficient (*S*_11_) for each of the sensors measured in the 2% saline solution. For the sake of completeness, the simulated result is also shown. The value of *S*_11_ is below −10 dB over the frequency range of interest, indicating that most of the power is radiated or absorbed by the antenna but not reflected back to the source. Discrepancies between the responses of the two sensors as well as the simulations and measurements are observed. These discrepancies can be mostly attributed to the sensitive construction process of the Cassiopeia. As described in [10], this sensor is composed of several metallic and plastic pieces which have to be machined and assembled. In addition, a cement material is poured into the sensor aperture which needs to fill tight spaces. Between machining variation of plastic material and the difficulty in controlling the cement filling process, construction discrepancy between sensors is to be expected. We also note that the discrepancy between simulated and measured response increases for the second half of the frequency band, and this is due to inexact material properties used in simulation. Specifically, the sensor utilizes Eccostock HiK materials for which models of the dielectric properties over the entire frequency band of interest are not available.

**Figure 8:**
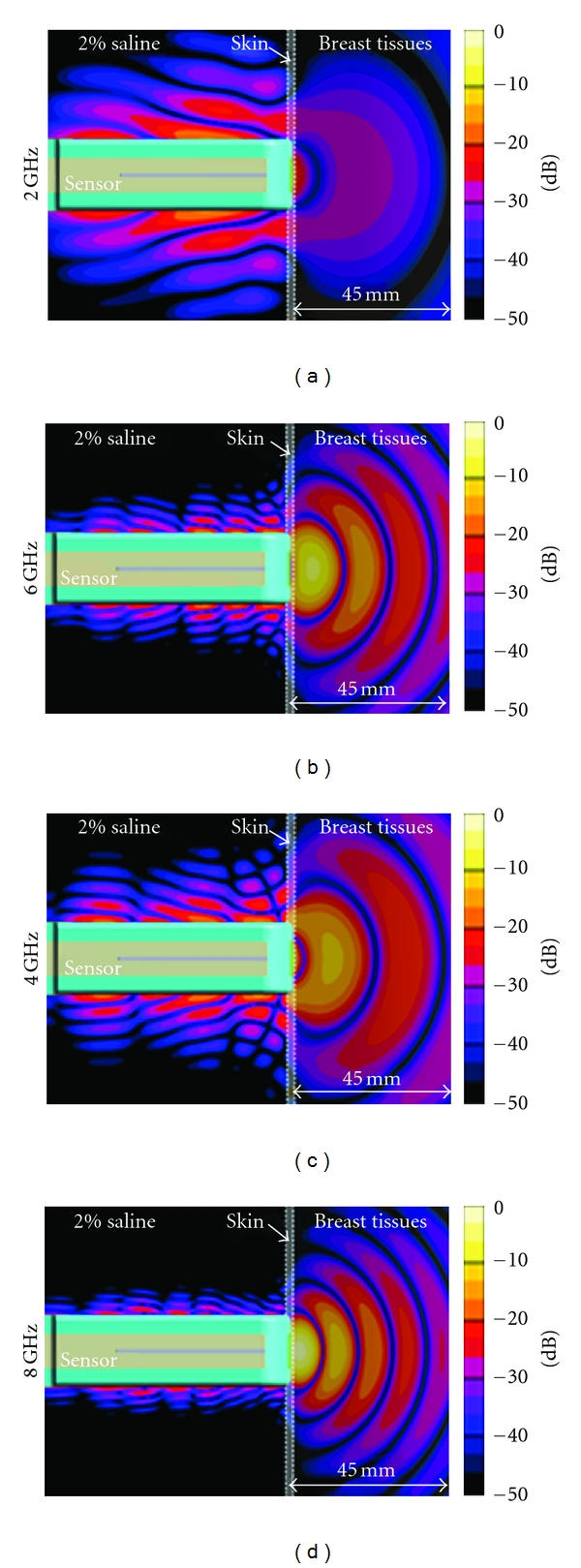
Electric field radiated by the antenna in realistic breast tissues while immersed in 2% saline. All field intensities are normalised to identical input power. The side-on view is shown (i.e., the cross-section through the cylindrical breast model is along its long axis).

Figure 7 suggests that, as per the results presented in [10], the Cassiopeia can reasonably operate in conductive media. However in a high-permittivity liquid, such as the 2% saline, the antenna starts to behave as a leaky wave structure, especially at lower frequencies (below 3 GHz). This is illustrated in Figure 8, which presents the simulated radiation behaviour when the antenna contacts the breast model, as in [9], consisting of a skin layer filled with a material representing low-loss tissues. At 2 GHz, the structure radiates in the vertical direction and the intensity of radiation in the breast tissues is quite low, considering that the breast tissues are considerably less lossy than the immersion medium. This behaviour is the result of the antenna becoming a leaky wave structure in high-permittivity media. At higher frequencies (4, 6, 8 GHz), the leaky wave effect is significantly reduced as the field is more confined in the antenna structure. Based on this simulated result, significantly lower transmission measurements are expected in the lower-frequency band.

**Figure 9:**
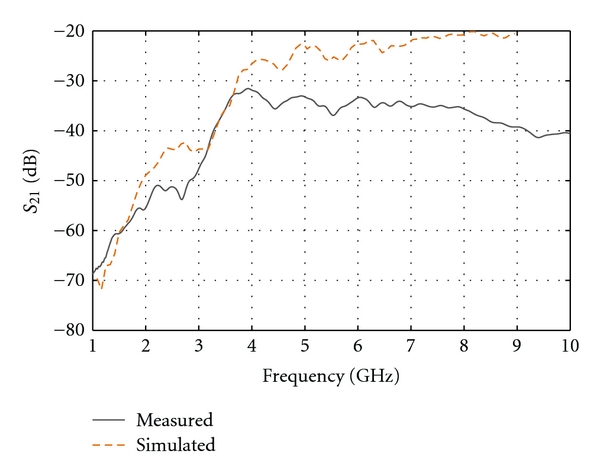
Measured and simulated transmission through the low-loss breast model (100 mm diameter).

Figure 9 presents the measured and simulated transmission coefficient obtained with the low-loss breast model. At the lower frequencies, a very low transmission magnitude is noted, as expected from Figure 8. However, the transmission level appears to stabilize at 4 GHz and above. We note that, at the higher frequencies, the simulated data predict a stronger transmission than observed in practice. Again, differences in the Eccostock HiK material properties between measurements and simulations are the main source of this disagreement, especially the conductivity which, in practice, significantly increases with frequency. Nevertheless, the main observation is that the measurement system demonstrates the ability to measure transmitted signals over the frequency band of interest.

**Figure 10:**
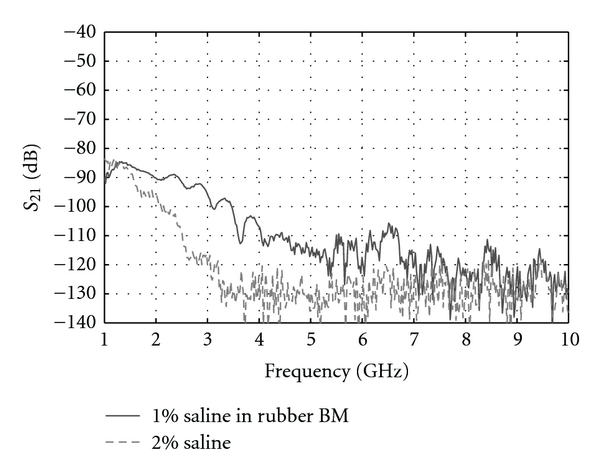
Transmission measurement through a rubber membrane containing a 1% saline solution (“high-loss breast model”) and with 2% saline filling the region between the two sensors positioned with the same separation distance (100 mm).

The transmission coefficients measured through the high-loss breast model are presented in Figure 10 and demonstrate the ability of our measurement system to measure very weak transmitted signals. More precisely, one may observe that the transmission coefficient measured, while the breast model presented is easily distinguished from the measurement taken at the same sensor separation without the breast present until both signals reach the noise floor. In other words, this result suggests that these weak recorded signals actually propagate into the breast model and not in the immersion medium or the tank structure.

### 3.2. Property Estimation

**Figure 11:**
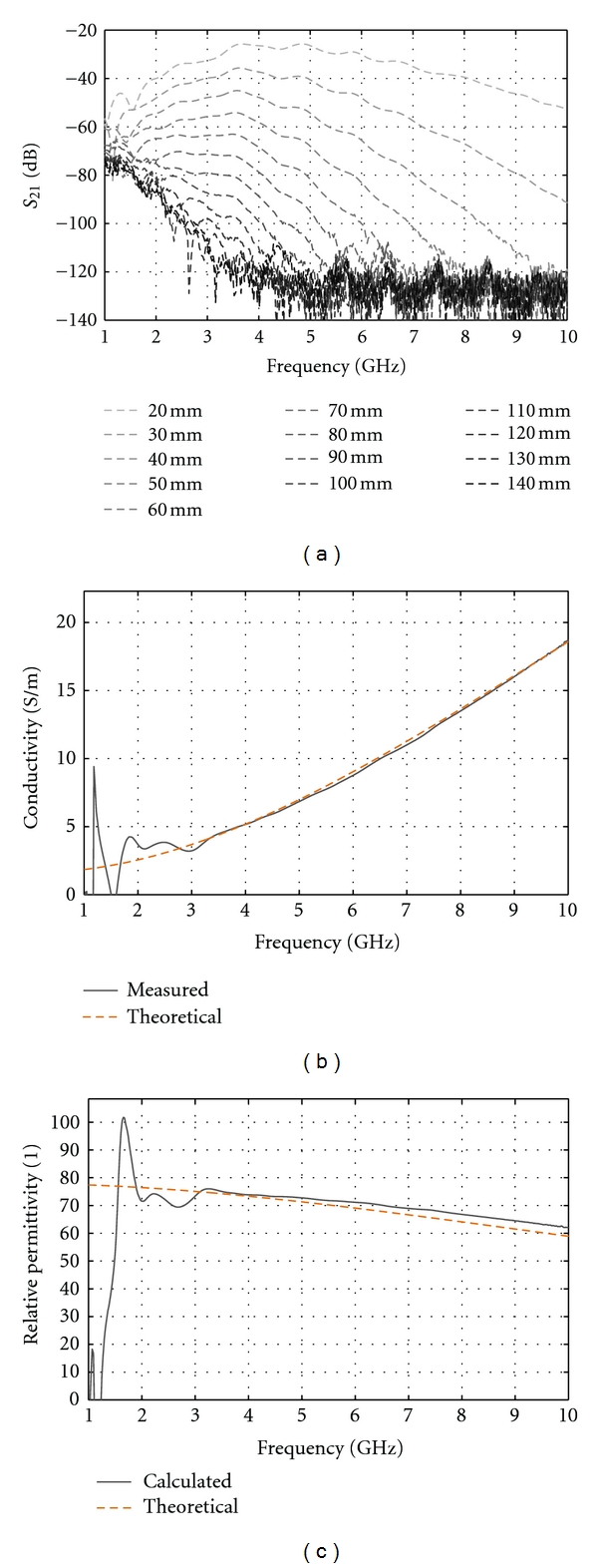
Measurement (a) taken with different separation distances of the sensors and the tank containing only a 1% immersion liquid. Conductivity (b) and relative permittivity (c) calculated using (2) and (3) and compared with theoretical values based on [12].

In order to validate the property estimation technique, measurements with the tank containing only a 1% saline immersion medium are used. This lower-loss medium was chosen instead of the 2% saline in order to measure signals over a wider-frequency bandwidth. The transmission measurements for several distances are shown in Figure 11(a), while the calculated permittivity and conductivity, using the two shortest separation distances along with (2) and (3), are shown in Figures 11(b) and 11(c). One can observe that the estimation is not very accurate below 3 GHz. At these frequencies, the poor radiation behaviour of the antenna, translated in a very low coupling efficiency, contributes to the error in results. However, above 3 GHz, the calculated values are very close to the theoretical ones, confirming that the assumptions made to simplify estimation of average properties are valid for this simple case.

In summary, the results presented in this section demonstrate that the Cassiopeia antenna, immersed in 2% saline, exhibits low *S*_11_ over a very wide band, and that radiated field behaviour improves with frequency (related to leaky wave behaviour at lower frequencies). The work with two sensors demonstrates that the system is capable of detecting signals transmitted through objects with low and high loss characteristics over the entire frequency range of interest. The approach to estimating average properties is verified for a simple scenario, showing that reasonable estimates are obtained from 3 to 10 GHz.

## 4. Volunteer Study

To gain insight into the maximum frequency at which signals transmitted through the breast may be detected, a study of 10 volunteers has been performed. The description of the study is followed by presentation of the measured data. Finally, the average property estimation is applied to the measured data, providing mixed results.

### 4.1. Recruitment and Protocol

This study has been approved by the Conjoint Health Research Ethics Board (CHREB) of the University of Calgary, (ID 23244). The volunteers were recruited through billboard posters and by word of mouth. To ensure good contact with the sensors, the breasts must extend far enough into the measurement tank. Therefore, one of the criteria for participation in the study was a minimum “C” cup size. The age of the volunteers was recorded; however, no additional information on breast tissue composition was available (e.g., X-ray mammograms or other breast imaging results). The volunteers are referred to by their ages.

Measurements took place in a research laboratory with a registered nurse helping the volunteers to be positioned on the machine. A research engineer controlled the data acquisition (from behind a privacy screen), while the nurse adjusted the separation distance between the sensors. The left and right breasts were examined and a total of four measurements were taken for each breast. First, the sensors were positioned to contact the breast skin and an initial measurement was recorded. Then, one of the sensors was moved 5 mm toward the breast and a second measurement was recorded. The third measurement was recorded after moving the second sensor 5 mm closer to the breast. Finally, the first sensor was brought another 5 mm closer before the fourth measurement was recorded. Therefore, a total of 4 measurements (each at a different separation distance) was taken for each breast. This procedure was successfully realized for all of our volunteers without any discomfort reported.

### 4.2. Measured Results

**Figure 12:**
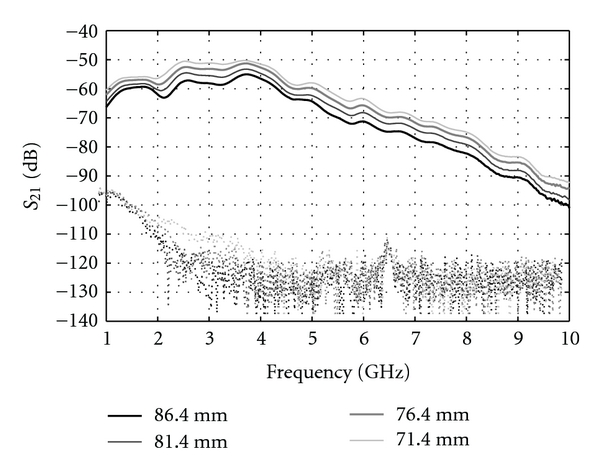
Transmission magnitude for the 35-year-old volunteer, left breast with different separation distances between the sensors. The dashed curves correspond to the transmission measured for the same distances but without the breast present.

**Figure 13:**
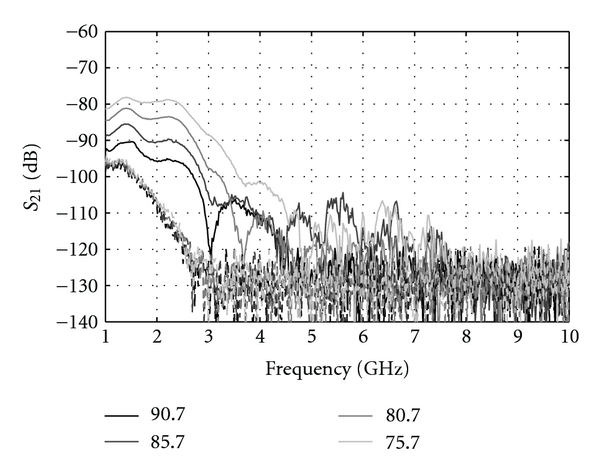
Transmission magnitude for the 55-year-old volunteer, left breast with different separation distances between the sensors. The dashed curves correspond to the transmission measured for the same distances but without the breast present.

Volunteers between 21 to 65 years of age participated in our study. As expected, we have observed considerable variation in signal attenuation due to variation in breast tissues between individuals. For example, in the case of a 35-year-old individual (Figure 12), signals were recorded for the entire frequency band of interest. While for a 55-year-old volunteer (Figure 13), the transmission is weaker, and no significant signals are recorded after 4 GHz. We note that results for each patient typically exhibit similar characteristics, namely, increased transmission with decreased separation between sensors as well as decreased transmission with frequency.

**Figure 14:**
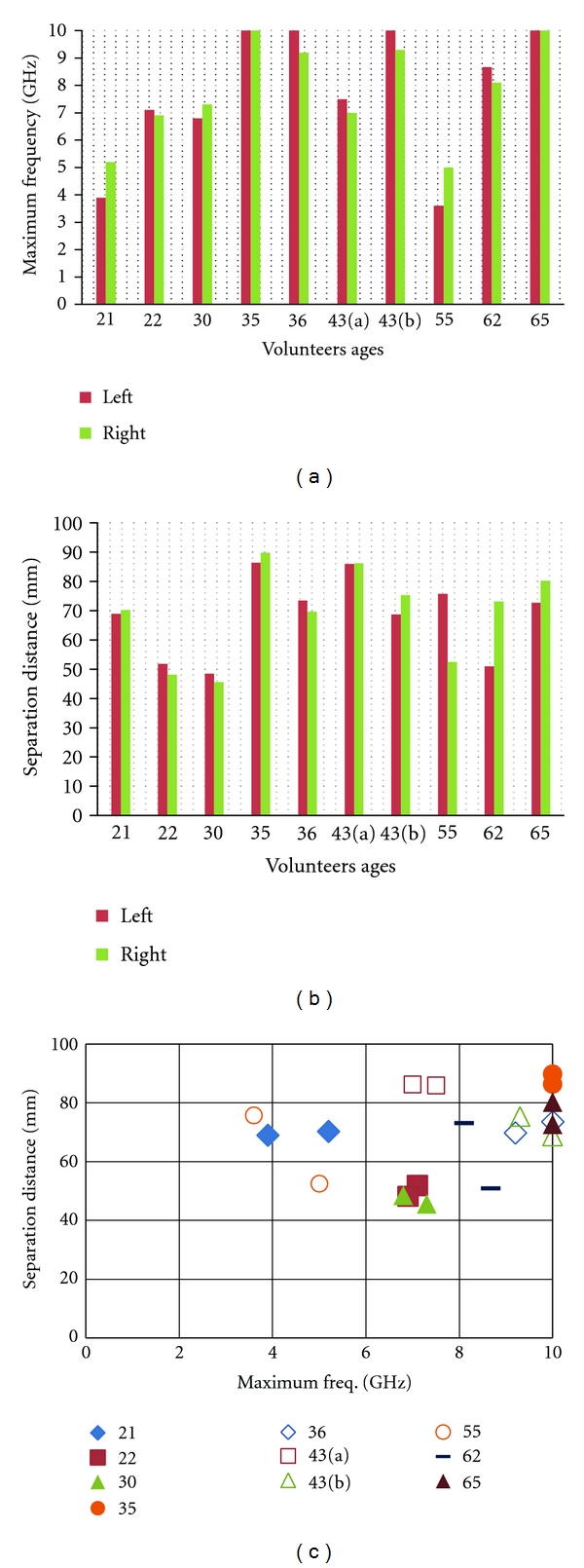
Measurement results: (a) maximum frequency and (b) separation distances in function of volunteer ages. (c) separation distance as a function of maximum frequency for each volunteer breast.

In order to compare the attenuation level between each case, the last frequency point for which the transmitted signal achieves a 20 dB signal-to-noise ratio (SNR) is used as metric. For this specific measurement system, the 20 dB SNR translates to a transmission coefficient level of −100 dB. This point is referred to the “maximum frequency”, and the observed values are plotted in Figure 14(a). The maximum frequency is determined for the shortest separation distance between sensors that permits this measurement to be collected. The corresponding separation distances are also given in Figure 14(b).  Figure 14(c) investigates the relation between separation and maximum frequency, showing no correlation. This confirms that the variations in maximum frequency are related to differences in breast tissue composition among our volunteers.

Figure 14(a) shows that, while the maximum frequency for the patients varies between 3.5 to 10 GHz (measurement limit), a certain symmetry between the left and right breasts for individual patients is observed. Only the 21-and 55-year-old volunteers show larger differences between breasts. However, in the case of the 55-year-old individual, the separation distances (Figure 14(b)) are quite different. When maximum frequencies for similar distances are compared, better symmetry is observed. This suggests that the variation seen in the maximum frequency for the 55-years-old volunteer is due to difference in separation distances. It is also observed that the separation distances for our 62-year-old volunteer are significantly different between the left and right breast; however, good symmetry in the maximum frequency is still noted. This volunteer had previous surgery on her left breast, so perhaps changes in tissue properties (e.g., scar tissue) result in similar maximum frequencies at different separation distances.

While not sufficient for generating statistics, the measurements do suggest the possibilities of using signals up to 10 GHz to image breast tissues, depending on breast composition. At the same time, the measured transmission levels are specific to our system. Sensors with higher gain and measurement equipment with better dynamic range would increase the overall sensitivity. On the other hand, it was practically observed that a slight decrease of the transmission path length can significantly increase the signal intensity, which makes moderate breast compression an appealing option. For example, in Figure 13, more than 10 dB in transmission is gained with a 10 mm decrease of separation between sensors.

### 4.3. Property Estimates

For each patient, the property estimation technique is applied to the 6 combinations of data available from the 4 measured transmission coefficients; the results are averaged to produce a single estimate. This averaging is performed to limit the effect of possible outliers in the measured data. The accuracy of the result is difficult to assess since the ground truth remains unknown. However, based on the observed attenuation, preliminary estimates of tissue properties and/or breast composition can be hypothesised.

**Figure 15:**
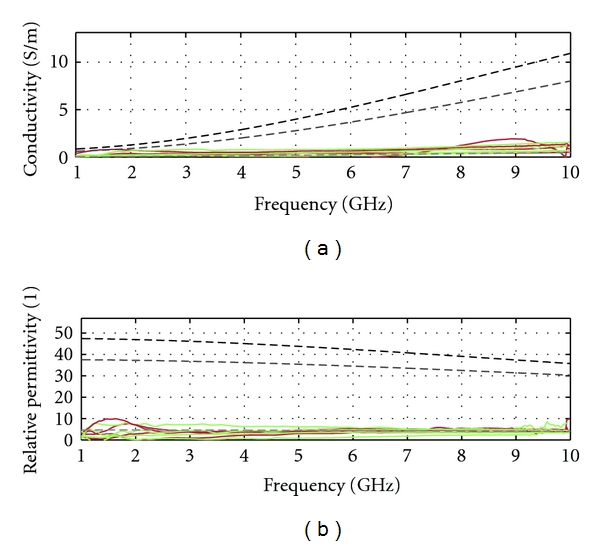
Estimation of breast tissue electrical properties (conductivity in (a) and permittivity in (b)) for the 35-, 36-, 43(a)- and 65-year-old volunteers. Groups 1, 2, and 3 refer to the glandular, transition, and fatty categories of tissues defined in [1].

For example, volunteers 35, 36, 43(a), and 65 years of age have maximum frequency for transmission reaching 10 GHz with a significant separation distance between sensors. This suggests that these patients have predominantly fatty breast tissues (tissue group 3). The estimated properties for these volunteers' breasts are shown in Figure 15, converging toward the group 3 model as hypothesized.

**Figure 16:**
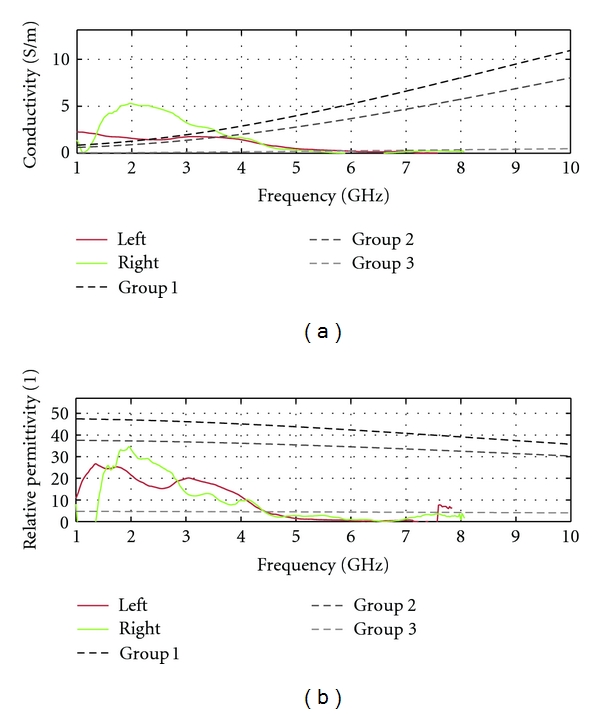
Estimation of breast tissues electrical properties (conductivity in (a) and permittivity in (b)) for the 30-year-old volunteer. Groups 1, 2, and 3 refer to the tissue categories defined in [1].

For breasts that likely have more significant glandular tissue composition (i.e., lower maximum frequencies), the average property estimation is unfortunately not as successful. For example, the permittivity estimates show significant variations with frequency, and the permittivity curve usually does not follow the expected Cole-Cole model shape. The conductivity usually behaves close to expected; however, some results show unexpectedly high conductivities or conductivity that decreases with frequency. To illustrate these observations, estimated properties for our 30-year-old volunteer are shown in Figure 16. Several factors may be the source of these behaviors. First, heterogeneous breasts are very likely to generate multipath, as signals may travel through fatty tissue or glandular tissue, hence arriving at different times at the sensor. This multipath makes our simple propagation model inadequate. Secondly, our estimation technique assumes that the breast tissues deform homogeneously. This means that the changes in the transmission coefficient are related to the entire breast and not local volumes. It is known that the elastic modulus values are not consistent across different tissue types [15], which means that different tissues deform differently. As a result, the change in transmission can arise from local deformation, instead of the assumed homogeneous deformation. Therefore, alternative techniques to estimate average properties are required for breasts that are not predominantly fatty.

## 5. Conclusion

The prototype system reported in this paper is designed specifically to measure transmitted signals through the breast. Antennas designed to contact the breast during microwave measurement are adapted to measure transmitted signals. Two antennas are placed on opposite sides of the breast, while immersed in saline. Through operation in a lossy medium, signals traveling along alternate paths are attenuated, such that the signal traveling directly through the breast is expected to have a predominant effect on the measurement. As the system is aimed at determining appropriate frequencies of operation, it operates over a wide band (1–10 GHz). Therefore, the bandwidth and approach to measurement differ from previously reported prototype systems aimed at microwave imaging (e.g., [7, 8]). In particular, the system is designed to enable transmission measurements through the entire breast over a frequency range from 1 to 10 GHz. We note that microwave tomography systems also involve measurement of signals transmitted through the breast (as well as a lossy immersion liquid); however, a maximum measurement frequency of 3 GHz has been used in work with patients. Therefore, the system reported in this paper permits exploration of transmitted signals over a higher-frequency band.

The system is deployed in a study of 10 volunteers with unknown breast composition. For each volunteer, four measurements are collected per breast, with separation between sensors adjusted for each measurement. The maximum frequency at which transmitted signals are reliably measured is noted, and significant variation in this frequency is observed for the 10 volunteers. There is, however, similarity between the maximum frequencies observed for the right and left breasts. In addition, the results of our measurements suggest that the use of frequencies up to 10 GHz is realistic. However, for denser breasts, this upper limit is likely considerably reduced. In such cases, the image reconstruction scheme (whether tomographic or radar-based) could be adapted to use only the relevant frequency band. As a result, the image resolution would likely be patient-dependant in the same way as image contrast depends on breast density with X-ray mammography. It was also practically observed that the use of moderate compression and sensors that contact the breast significantly improves SNR. Therefore, results of this study suggest that the data collected with this system provide unique insights into microwave measurements of the breast.

Finally, an approach to estimating the average properties of breast tissues has been introduced, and reasonable results are obtained for volunteers with suspected primarily fatty breasts. This method is based on several assumptions that do not appear to hold for volunteers that are suspected to have greater glandular tissue content. Therefore, future work includes developing average property estimation techniques that are effective for a wider range of breast compositions. In addition, a new waveguide like UWB sensor is being developed to avoid leakage into the surrounding saline medium and which improves the coupling efficiency with the breast tissues, especially at lower frequencies. For further investigation, a second study is planned that also includes clinical breast imaging in order to give insight into the breast composition and aid in interpreting results.
